# A randomized controlled trial protocol of the cardiovascular safety and efficacy of liraglutide in the treatment of type 2 diabetes

**DOI:** 10.1097/MD.0000000000023948

**Published:** 2021-01-22

**Authors:** Ying Liang, Hua Meng, Ruiyu Li, Jianbin Yang, Jingchao Jia, Yongli Hou

**Affiliations:** a Department of Cardiology, Changhai Hospital of Naval Military Medical University, Shanghai 200438; b Department of Medicine, People's Hospital of Ningxia Hui Autonomous Region, Yinchuan 750002; c Department of Chinese Medicine, The Second Affiliated Hospital of Xingtai Medical College, Hebei 054000; d Department of Pharmacy, Xingtai People's Hospital, Hebei 054001; e Department of Research Office, The Second Affiliated Hospital of Xingtai Medical College, Hebei 054000, China.

**Keywords:** glucagon-like peptide 1, liraglutide, randomized, study protocol, type 2 diabetes

## Abstract

**Background:**

Recently, many clinical experiments have evaluated the influences of liraglutide in the treatment of type 2 diabetes. However, the outcomes of these studies are inconsistent, and the number of high-quality prospective trials that conducted to assess the cardiovascular safety is limited. Hence, for this research, it was implemented for the assessment of the cardiovascular effectiveness and safety of liraglutide in type 2 diabetes patients.

**Methods:**

This research was a 26-week active controlled and randomized trial. Our research protocol follows the guidelines of Good Clinical Practice issued via the Helsinki Declaration and International Conference on Coordination. All the patients will receive the written informed consent in order to involve in our clinical experiment. The participants with type 2 diabetes aged from 18 years to 80 years, patients with 45.0 kg/m^2^ body-mass index or less, and with glycosylated hemoglobin of 7.5 to 10.0 percent, and received metformin (daily 1500 mg or more) for 3 months or longer were eligible. All the patients were randomized to 1 of 2 interventions (in the ratio of 1:1): liraglutide placebo once daily (blinded) and liraglutide once daily (blinded), respectively, both combined with the glimepiride and metformin (open-labeled). For the efficacy variable, the major endpoint was the baseline glycated hemoglobin change after treating for 26 weeks. The secondary end points involved: the percentage of participants who achieved the goals of postprandial blood glucose, fasting blood glucose, and glycosylated hemoglobin; the changes of mean postprandial blood glucose, fasting blood glucose, and the body weight, pancreatic B-cell function index, and changes in blood pressure and insulin resistance assessed by homeostasis model.

**Conclusions:**

For this research, the limitations involve the short trial period and the limitation of glimepiride in some countries, thus excluding the maximum doses of glimepiride.

**Trial registration:**

This study protocol was registered in Research Registry (researchregistry6306).

## 1 Introduction

Type 2 diabetes is a kind of progressive multisystem disease. Patients with type 2 diabetes have insulin resistance, different degrees of decreased function of β-cell, and inability to inhibit the postprandial secretion of glucagon. It is related to latently devastating complications and a range of complications.^[1–3]^ The present treatments do not control the blood glycemia in the long term owing to they do not address the problem of impaired function of beta cell and do not have a positive impact on cardiovascular or weight problems related to disease. Furthermore, such treatment generally involves titration protocols and complex treatment that may enhance the risk and side effects of hypoglycemia, for instance, weight gain and edema.^[4,5]^

Glucagon-like peptide 1 (GLP-1) is an insulin-stimulating hormone generated in the gut, it can reduce the secretion of glucagon, stimulate the glucose-dependent endogenous insulin secretion, slow the gastric peristalsis and empty-out, and then decrease the appetite and intake of food.^[6–9]^ In addition, in the animal models, the native GLP-1 can stimulate the proliferation of β-cell and promote the inhibition of apoptosis in the vitro, this may increase the function and mass of β-cell.^[10]^ Liraglutide, a kind of once-daily human analogue of GLP-1, has 97 percent homology with the linear amino-acid sequence of human GLP-1. The half-life of liraglutide after subcutaneous injection is 13 hours, which can generate 24-hour control of blood glucose. Liraglutide has been confirmed to reduce the levels of glucose, and many former clinical experiments have indicated that liraglutide is related to blood pressure and mild weight loss.^[11–13]^

Recently, many clinical experiments have evaluated the influences of liraglutide in the treatment of type 2 diabetes. However, the outcomes of these studies are inconsistent, and the number of high-quality prospective trials that conducted to assess the cardiovascular safety is limited.^[14–17]^ Hence, for this research, it was implemented for the assessment of the cardiovascular effectiveness and safety of liraglutide in type 2 diabetes patients.

## 2 Materials and methods

### 2.1 Trial design

This research was a 26-week active controlled and randomized trial. Our research protocol follows the guidelines of Good Clinical Practice issued via the Helsinki Declaration and International Conference on Coordination. The study protocol was approved by the ethics committee of the Second Affiliated Hospital Of Xingtai Medical College. All the patients will receive the written informed consent in order to involve in our clinical experiment. The research protocol was registered in Research Registry, with the number researchregistry6306.

### 2.2 Participants

The participants with type 2 diabetes aged from 18 years to 80 years, patients with 45.0 kg/m^2^ body-mass index or less, and with glycosylated hemoglobin of 7.5 to 10.0 percent, and received metformin (daily 1500 mg or more) for 3 months or longer were eligible. The major criteria for exclusion contained: use of any anti-hyperglycemic drug treatment other than the metformin within 3 months after trial; current use of any drugs other than the metformin that may have impact on the blood glucose; repeated severe hypoglycemia or unclear awareness of hypoglycemia; patients with contraindications of experimental drugs; impairment of liver or renal function; cancer or cardiovascular diseases of clinical significance.

### 2.3 Randomization and interventions

The eligible patients would be randomly divided into 2 groups through applying the random numbers generated by computer. Random numbers were kept in the opaque sealed envelopes, all the patients were asked to choose a random envelope for the determination of treatment group. All the patients were randomized to 1 of 2 interventions (in the ratio of 1:1): liraglutide placebo once daily (blinded) and liraglutide once daily (blinded), respectively, both combined with the glimepiride and metformin (open-labeled). The liraglutide placebo and liraglutide were offered via the hospital central pharmacy. The participants, researchers, and the study monitors were not informed at any time about the treatment of the placebo and liragtide groups.

### 2.4 Dosing of interventions

After the randomization, in liraglutide group, the patients received an incremental dose of 0.6 mg once a day for 2 weeks, increasing by 0.6 mg per week to reach 1.8 mg ultimate daily dose at the end of the second week; the injections of daily placebo was matched. After a period of dose escalation for 2 weeks, the dose of liraglutide was fixed for 24 weeks. Experimental drugs are injected subcutaneously into the upper arm, thigh, or abdomen through utilizing the prefilled pen device. Injections can be given at any time of day. The patients were encouraged to receive the injection of liraglutide daily for the same period of time. After the randomization, the dose of glimepiride could be reduced from 4 mg to 2 mg if the hypoglycemia and adverse events were required.

### 2.5 Outcomes

For the efficacy variable, the major endpoint was the baseline glycated hemoglobin change after treating for 26 weeks. The secondary end points involved: the percentage of participants who achieved the goals of postprandial blood glucose, fasting blood glucose, and glycosylated hemoglobin; the changes of mean postprandial blood glucose, fasting blood glucose, and the body weight, pancreatic B-cell function index, and changes in blood pressure and insulin resistance assessed by homeostasis model. The safety variables contained episodes of hypoglycemic on the basis of blood glucose levels (less than 3.1 mmol/l), main adverse events of cardiovascular (for instance, stroke, and acute myocardial infarction), pulse and tolerability, and liraglutide antibodies (containing the neutralizing and cross-reacting antibodies). At the same time, electrocardiogram, vital signs, hematological, and biochemical indexes involving calcitonin were monitored.

### 2.6 Statistical analysis

The standard deviation and mean are the descriptive statistics. The normality of all the data was detected via the Kolmogorov–Smirnov test. The paired sample *T* test was utilized to calculate the difference of value before and after the treatment. For the 2 groups, their variances were compared with Student's *T* test. SPSS 16.0 was applied for analysis (SPSS Inc, Chicago, Illinois), and the level of significance is still at *P* < .05.

## 3 Discussion

In recent years, a number of clinical trials have evaluated the influence of liraglutide in type 2 diabetes patients. However, the outcomes of these studies are inconsistent, and the number of high-quality prospective trials that conducted to assess the cardiovascular safety is limited. Hence, for this research, it was implemented for the assessment of the cardiovascular effectiveness and safety of liraglutide in type 2 diabetes patients. The advantages of this research involve the inclusion of oral hypoglycemic agents and placebo optimized prior to randomization, at the aim of reducing the risk of hypoglycemia. For this research, the limitations involve the short trial period and the limitation of glimepiride in some countries, thus excluding the maximum doses of glimepiride.

## Author contributions

**Conceptualization:** Jianbin Yang, Jingchao Jia.

**Data curation:** Ying Liang, Hua Meng, Ruiyu Li.

**Formal analysis:** Ying Liang, Hua Meng, Ruiyu Li.

**Funding acquisition:** Yongli Hou.

**Investigation:** Ying Liang, Hua Meng, Ruiyu Li.

**Methodology:** Jianbin Yang.

**Project administration:** Yongli Hou.

**Resources:** Ruiyu Li, Yongli Hou.

**Software:** Jingchao Jia, Ruiyu Li.

**Supervision:** Yongli Hou.

**Validation:** Ruiyu Li, Jingchao Jia.

**Visualization:** Jingchao Jia.

**Writing – original draft:** Ying Liang, Hua Meng.

**Writing – review & editing:** Yongli Hou, Ruiyu Li.
